# Mechanistic Insight of Sensing Hydrogen Phosphate in Aqueous Medium by Using Lanthanide(III)-Based Luminescent Probes

**DOI:** 10.3390/nano11010053

**Published:** 2020-12-28

**Authors:** Jashobanta Sahoo, Santlal Jaiswar, Pabitra B. Chatterjee, Palani S. Subramanian, Himanshu Sekhar Jena

**Affiliations:** 1 Inorganic Materials and Catalysis Division, Central Salt and Marine Chemicals Research Institute (CSIR-CSMCRI), Bhavnagar, Gujarat 364 002, India; s.jashobanta@gmail.com; 2 Academy of Scientific and Innovative Research (AcSIR), CSIR-CSMCRI, Bhavnagar, Gujarat 364 002, India; pbchatterjee@csmcri.res.in; 3 Department of Chemistry, Hindol College, Khajuriakata, Higher Education Department, State Government of Odisha, Bhubaneswar, Odisha 751001, India; 4 Discipline of Marine Biotechnology and Ecology, CSIR-CSMCRI, Bhavnagar, Gujarat 364 002, India; santlal@csmcri.res.in; 5 Analytical Discipline and Centralized Instrument Facility, CSIR-CSMCRI, Bhavnagar, Gujarat 364 002, India; 6 Department of Chemistry, Ghent University, Krijgslaan 281-S3 B, 9000 Ghent, Belgium

**Keywords:** lanthanides, luminescence, nitrogen-rich ligand, phosphate sensing, quenching

## Abstract

The development of synthetic lanthanide luminescent probes for selective sensing or binding anions in aqueous medium requires an understanding of how these anions interact with synthetic lanthanide probes. Synthetic lanthanide probes designed to differentiate anions in aqueous medium could underpin exciting new sensing tools for biomedical research and drug discovery. In this direction, we present three mononuclear lanthanide-based complexes, EuLCl_3_ (**1**), SmLCl_3_ (**2**), and TbLCl_3_ (**3**), incorporating a hexadentate aminomethylpiperidine-based nitrogen-rich heterocyclic ligand **L** for sensing anion and establishing mechanistic insight on their binding activities in aqueous medium. All these complexes are meticulously studied for their preferential selectivities towards different anions such as HPO_4_^2−^, SO_4_^2−^, CH_3_COO^−^, I^−^, Br^−^, Cl^−^, F^−^, NO_3_^−^, CO_3_^2−^/HCO_3_^−^, and HSO_4_^−^ at pH 7.4 in aqueous HEPES (2-[4-(2-hydroxyethyl)piperazin-1-yl]ethanesulfonic acid) buffer. Among the anions scanned, HPO_4_^2−^ showed an excellent luminescence change with all three complexes. Job’s plot and ESI-MS support the 1:2 association between the receptors and HPO_4_^2−^. Systematic spectrophotometric titrations of **1**–**3** against HPO_4_^2−^ demonstrates that the emission intensities of **1** and **2** were enhanced slightly upon the addition of HPO_4_^2−^ in the range 0.01–1 equiv and 0.01–2 equiv., respectively. Among the three complexes, complex **3** showed a steady quenching of luminescence throughout the titration of hydrogen phosphate. The lower and higher detection limits of HPO_4_^2−^ by complexes **1** and **2** were determined as 0.1–4 mM and 0.4–3.2 mM, respectively, while complex **3** covered 0.2–100 μM. This concludes that all complexes demonstrated a high degree of sensitivity and selectivity towards HPO_4_^2−^.

## 1. Introduction

**Scheme 1:**
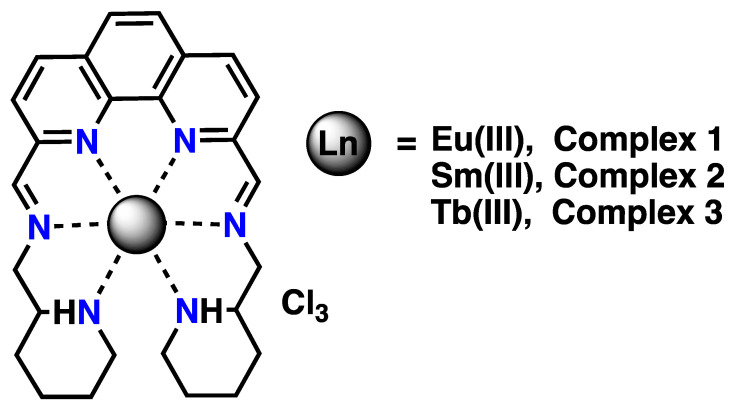
2,9-Bis(aminomethylpiperidine)-1,10-phenanthrolene (**L**) and its Ln(III) complexes **1**, **2**, and **3**.

Inorganic phosphates, the charged anions of phosphoric acid such as [H_2_PO_4_]^−^, [HPO_4_]^2−^, and [PO_4_]^3−^, are essential components during the synthesis of DNA/RNA and phospholipid membrane [1]. Further, their influence in the metabolic process in human, plant, and animal cells are inevitable. Sensing of phosphate draws special attention [2,3,4,5,6,7,8,9,10,11,12,13] due to its biological role as polyphosphate, and hyper- and hypophosphatemia in Chronic Kidney Disease (CKD) patients [14]; energy source through dephosphorylation [15] of ATP, ADP, AMP, and PPi; and reverse polycondensation to form polyphosphates. Various methods were developed for the determination of phosphates in fertilizers, plants, natural waters, and other environmental samples [16,17,18]. Generally, serum phosphates are measured based on a photometric approach using ammonium phosphate, which forms a chromogenic complex with inorganic phosphates (Pi) [19]. However, the search for new receptors with selective response to phosphates remains active behind many challenges. Moreover, with phosphates being important bioanlytes [20,21,22,23,24,25,26,27,28,29], varieties of colorimetric sensors [30,31,32,33,34,35] and fluorosensors [36,37,38] were reported for their detection. Among these, luminescent lanthanide [20] complexes gained significant attention due to their potential applications in clinical diagnosis, biomarkers [39,40], MRI contrast agents [41,42,43,44,45,46,47], screening of drugs, etc. Parker et al. reported Eu(III) and Tb(III) tetra-azaphenylene complexes for the detection of phosphates in live cells [48,49]. It is important to note that the concentrations of phosphate vary significantly in inter- and intracellular environments of human cells, ranging from 0.15 to 1.3 mM [50,51,52]. Among the various lanthanide complexes reported so far in the literature, Eu-Tc [53,54] was recognized as an efficient probe for phosphates due to its lower detection limit (LOD = 3 μmolL^−1^). Moreover, there are many intracellular processes, where the concentrations of phosphate vary among different subcellular compartments present therein [55]. Therefore, a highly sensitive and selective probe which can detect phosphate at a considerably low concentration is very much required to investigate such intracellular processes. In this context, recently, we have reported a set of europium(III) and terbium(III) complexes, incorporating different hexadentate ligands which showed highly selective and efficient recognition of inorganic phosphates and nucleoside phosphates [56,57]. In this direction and as a part of our ongoing research, herein, we report a series of relatively simple, cheap, and water-soluble Ln(III) complexes **1**, **2**, and **3** (Scheme 1) (Ln = Eu, Sm, and Tb, respectively) using an aminomethylpiperidine-functionalized 1,10-phenanthrolene-based nitrogenous heterocyclic ligand **L** as the metal chelator. The anion-sensing ability of these hydrophilic rare-earth complexes (**1**–**3**) was explored and found high selectivity and sensitivity for hydrogen phosphate ions in HEPES (2-[4-(2-hydroxyethyl)piperazin-1-yl]ethanesulfonic acid) buffer at pH 7.4. Moreover, a mechanistic insight into the anion binding behavior of complex **1** was also explored in this work. 

## 2. Results and Discussion

**Figure 1:**
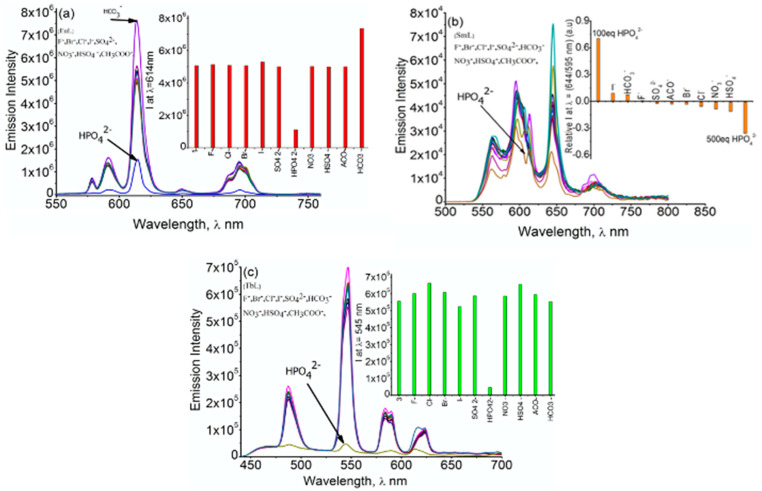
Emission spectra of (**a**) **1** (1 × 10^−5^ M), (**b**) **2** (2 × 10^−5^ M), and (**c**) **3** (4 × 10^−5^ M) upon the addition of various anions (100 equiv. for **1** and 10 equiv. for **3** in aqueous HEPES (2-[4-(2-hydroxyethyl)piperazin-1-yl]ethanesulfonic acid) buffer at pH = 7.4, λ_exi_ = 276 nm): in the case of **2**, spectra obtained after the addition of both 100 (cyano) and 500 equiv. (light brown) of HPO_4_^2−^ are overlaid. Insets: luminescence intensities of **1** and **3** at 614 and 545 nm, respectively, in the presence of different anions, while in the case of **2**, relative intensity ratios (644/595 nm) are plotted along the y-axis.

Schiff base ligand **L** was obtained by condensing 2,9-dialdehyde 1,10-phenanthroline and 2-(aminomethyl) piperidine. The characteristic azomethine peak at 8.25δ in ^1^H NMR (Figure S1) and ^13^C (Figure S2), DEPT 135° NMR (Figure S3) in combination with MS spectrum (Figure S4), CD spectrum (Figure S5) and IR spectra (Figure S6), confirms the formation of **L**. Treating **L** with the respective LnCl_3_ salt, the corresponding complexes EuLCl_3_**(1**), TbLCl_3_(**2**), and SmLCl_3_(**3**) were isolated as per the procedure. The formulation of each complex was confirmed from the ESI-MS analysis (Figure S7–S9). The emission spectra of these complexes were studied at 25 °C in aqueous HEPES buffer at pH 7.4 (Figure S10). All the complexes showed a significant red-shift of the emission spectra with respect to the emission profile of the free ligand **L**. Being luminescent in nature, we sought to investigate the excited state photophysical properties of **1**–**3** in the presence of various important anions. Complexes **1** and **3** showed characteristic luminescent bands at 614 nm and 545 nm, respectively, while complex **2** displayed two sensitive bands at 595 and 644 nm attributable for their metal centered emission. Although water functions as a luminescence quencher [58], all these complexes showed an intense luminescence in aqueous HEPES buffer at physiological pH at 25 °C. The effects of the addition of a range of anions such as hydrogen phosphate, sulfate, acetate, iodide, bromide, chloride, fluoride, nitrate, carbonate/bicarbonate, and bisulfate on the emission spectra of **1**–**3** is showed in Figure 1a–c. The bar diagrams, as insets in Figure 1a,c, show the changes in emission intensities of the hypersensitive peaks at 614 and 545 nm of complexes **1** and **3**, while Figure 1b depicts the changes in the ratio of the 644/595 nm hypersensitive peaks of complex **2** with the addition of various anions.

**Figure 2:**
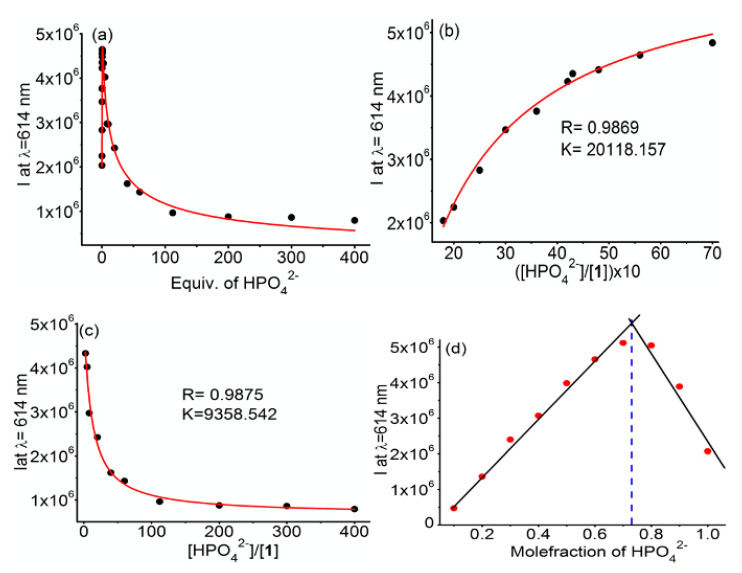
(**a**) Changes in emission maxima of **1** (1 × 10^−5^ M) upon gradual addition of HPO_4_^2−^ in aqueous HEPES buffer at pH = 7.4; (**b**) nonlinear curve fitting of the titration data as a function of HPO_4_^2−^ concentrations in the range 0.01–1 equiv. (luminescence enhancement part); (**c**) nonlinear curve fitting of the titration data as a function of HPO_4_^2−^ concentrations in the range 1–400 equiv. (luminescence quenching part), in which a factor of 10 was multiplied with enhancement to maintain the same intesnsity for both quenching and enhancement; and (**d**) Job’s plot analysis of mixtures of complex **1** with HPO_4_^2−^(*C*_complex **1**_ + *C*_HPO4_^2−^ = 1.0 μM) in aqueous HEPES buffer pH 7.0, indicating 1:2 complex formation (λ_emi_ = 614 nm).

Among the anions scanned, complex **1** illustrated a significant emission change with hydrogen phosphate and bicarbonate ions. While the addition of HCO_3_^−^ showed 14.7% luminescence enhancement, phosphate in contrast leads to luminescence quenching by 29% of the emission intensity of **1** (Figure 1a). In Figure S11, the emission spectra of **1** against varying concentrations of phosphates (0–400 equiv.) are shown. The emission intensity was found to increase (2.6-fold) initially in the range 0.01–1 equiv. of HPO_4_^2−^ (Figure 2a).

Upon further addition of HPO_4_^2−^ to the reaction mixture, a gradual decrease in the luminescence, at 614 nm, of receptor **1** was observed (Figure 2a). The changes in the luminescence intensities, as displayed in Figure 2a, can be attributed to the two distinct behaviours of **1** against HPO_4_^2−^. Therefore, the spectrometric titration (Figure S11) has offered two association constants. Figure 2a also displayed that the luminescence intensity of the emission maximum decreased to a constant level after the addition of 4 mM of phosphate. Therefore, it is evident from Figure 2a that **1** can be used to sense a wide range of phosphate concentrations. The analytical limit of detection (LOD) [59,60,61,62] of **1** for phosphate was calculated as 0.1 μM. Since the sensing of hydrogen phosphate showed nonlinear fitting in Figure 2b,c with one enhanced and the other quenching the luminescence intensity, we applied the nonlinear fit data point results by following Equations (1) and (2), [63,64,65,66] respectively, providing the binding constants *K*_1_ = 2.0 × 10^4^ M^−1^ (R = 0.9869), 1st part, attributable to luminescence enhancement and *K*_2_ = 0.94 × 10^4^ M^−1^ (R = 0.9875), 2nd part, associated to luminescence quenching.

(1) $$F=F_{0}+\frac{F_{max}-F_{0}}{2}+\{\left(1+\frac{\left[M\right]}{C_{L}}+\frac{1}{C_{L}K}\right)-\sqrt{{\left(1+\frac{\left[M\right]}{C_{L}}+\frac{1}{C_{L}K}\right)}^{2}-4\frac{\left[M\right]}{C_{L}}\}}\}$$

(2) $$F=F_{max}+\frac{F_{0}-F_{max}}{2}+\{\left(1+\frac{\left[M\right]}{C_{L}}+\frac{1}{C_{L}K}\right)-\sqrt{{\left(1+\frac{\left[M\right]}{C_{L}}+\frac{1}{C_{L}K}\right)}^{2}-4\frac{\left[M\right]}{C_{L}}\}}\}$$

where *F*_0_ is the luminescence intensity in the absence of hydrogen phosphate and *F_max_* is the luminescent intensity in the presence of HPO_4_^2−^, and *C_L_* and *K* are the concentration and binding constant of the complex, respectively. To find the association stoichiometry between complex **1** and HPO_4_^2−^, Job’s plot was performed (Figure 2d), which established 1:2 binding stoichiometry, i.e., [**1**:HPO_4_^2−^ = 1:2]_._ Further, a final confirmation regarding the abovementioned 1:2 stoichiometry was provided by ESI-MS (Figure S12), where a peak at *m*/*z* = 855.23 with 100% abundance was attributed to [EuL(HPO_4_)_2_(H_2_O) + 2Na^+^]·H_2_O (calcd. *m*/*z* = 855.11).

Unlike hydrogen phosphate, HCO_3_^−^ showed little enhancement in the emission of **1** (Figure 1a). The binding constant (*K*) was determined to be 1.2 × 10^3^ M^−1^ from the spectrometric titrations of **1** against increasing concentrations of HCO_3_^−^ (10 to 600 equiv.), as shown in Figure S13. Luminescence enhancement of **1**, observed in the addition of HCO_3_^−^, may occur due to chelate formation between the bicarbonate ion and europium (III) center by replacing the weakly bound inner sphere water molecules [67]. In Figure 1b, the luminescence response of **2** towards different anions is displayed. Among the four emission bands observed for **2**, the peaks at 595 nm (^5^G_5/2_→^6^H_7/2_) and 644 nm (^5^G_5/2_→^6^H_9/2_) were found to be hypersensitive [68,69,70].

**Figure 3:**
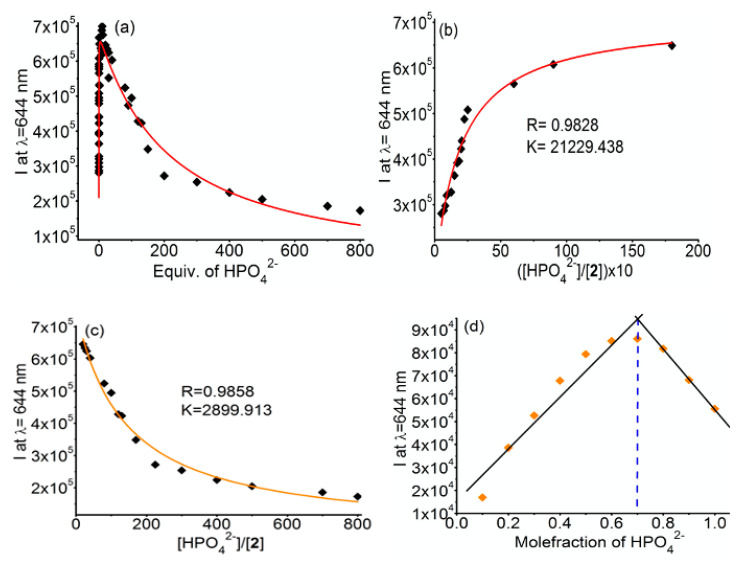
(**a**) Changes in emission maxima of **2** (4 × 10^−5^ M) upon gradual addition of HPO_4_^2−^ in aqueous HEPES buffer at pH = 7.4; (**b**) nonlinear curve fitting of the titration data as a function of HPO_4_^2−^ concentrations in the range 0.01–18 equiv. (luminescence enhancement part); (**c**) nonlinear curve fitting of the titration data as a function of HPO_4_^2−^ concentrations in the range 20–800 equiv. (luminescence quenching part); and (**d**) Job’s plot of complex **2** with HPO_4_^2−^(*C*_complex **2**_ + *C*_HPO4_^2−^ = 1.0 μM) in aqueous HEPES buffer pH 7.0 showing 1:2 complex formation (λ_emi_ = 644 nm).

Spectrophotometric titrations of **2** (Figure S14 and Figure 3a) against varying amounts of HPO_4_^2−^ ranging from 0.01 to 800 equiv. illustrated similar patterns as observed earlier in case of **1**. Interestingly, the initial luminescent enhancement of **2** was found up to 18 equiv. of phosphate addition and further increases in phosphate concentrations quench the luminescence of the resulting solution. Applying nonlinear fitting of the data points (Figure 3b,c), the respective binding constants (*K*_1_ and *K*_2_) were calculated to be 2.1 × 10^4^ M^−1^ (R = 0.9828), 1st part of luminescence enhancement and 2.9 × 10^3^ M^−1^ (R = 0.9858), 2nd part of luminescence quenching. The LOD was calculated to be 0.4 μM, a little higher than that observed for **1**. To derive the complex **2** to phosphate ratio, Job’s plot was performed, which clearly indicated 1:2 stoichiometry (Figure 3d). The respective positive ion ESI-MS (Figure S15) also confirmed the proposed 1:2 composition by depicting a molecular ion peak at *m*/*z* = 862.27 attributable to the formation of [Sm(L-2H)(HPO_4_)_2_ + 4Na^+^] (calcd. *m*/*z* = 862.05).

**Figure 4:**
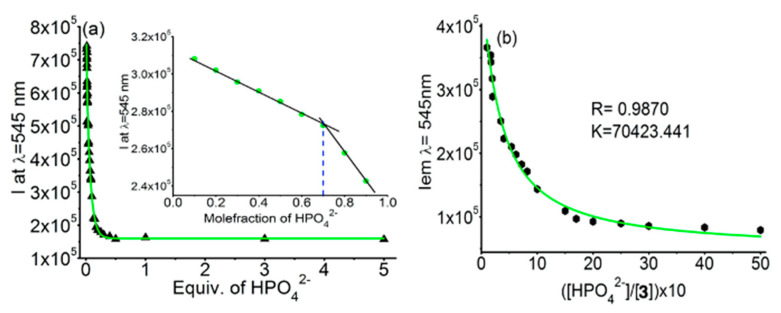
(**a**) Change in the emission spectra of **3** (2 × 10^−5^ M) upon the addition of HPO_4_^2−^ in aqueous HEPES buffer at pH = 7.4: the inset shows the Job’s plot of mixtures of complex **3** with HPO_4_^2−^(*C*_complex **3**_ + *C*_HPO4_^2−^ = 1.0 μM) in aqueous HEPES buffer pH 7.4 showing 1:2 complex formation. (**b**) Nonlinear curve fitting of luminescence intensities of **3** (2 × 10^−5^ M) as a function of HPO_4_^2−^ concentrations (λ_emi_ = 545 nm).

Screening complex **3** towards various anions (10 equiv.), shown in Figure 1c, illustrates again an excellent luminescent probe for HPO_4_^2−^ with superior selectivity and sensitivity. Upon the addition of HPO_4_^2−^, the emission intensity of **3** was reduced to 8%. Systematic spectrophotometric titration with an increasing concentration of hydrogen phosphate ions in the range 0.01–5 equiv. was performed (Figure 4a and Figure S16). Unlike, **1** and **2**, the luminescence intensity of **3** demonstrated a steady quenching process. The luminescence intensity was quenched continuously from the very beginning of HPO_4_^2−^ addition and at the 100 μM HPO_4_^2−^ concentration; the emission quenched completely and remained constant thereafter (Figure 4a). The LOD for complex **3** was derived as 0.2 μM. The binding constant was determined from nonlinear data fitting using the Equation (2), and the respective association constant (*K*) was found to be 7.0 × 10^4^ M^−1^ (R = 0.9870) (Figure 4b). A molecular ion peak at *m*/*z* = 803.56 (Figure S17) can be attributed to the generation of [TbL(HPO_4_)_2_ + Na^+^ + H^+^] (calcd. *m*/*z* = 803.45) in solution. Thus, all three complexes are established to bind phosphates in 1:2 stoichiometries.

Ligand **L** with its hexadentate nature fulfils six coordination sites of Eu(III), Sm(III), and Tb(III) in their respective complexes **1**, **2**, and **3**. The remaining sites at the metal centers were calculated by measuring the hydration states [11,71] (denoted hereafter by “*q*”) of **1**–**3**, adapting Equation (3). Based on the experimental results, the respective inner-sphere hydration numbers calculated for **1**–**3** are compiled in Table 1. Accordingly, complexes **1** and **2** are found to possess four coordinated water molecules while complex **3** accommodates only three water molecules, presumably due to the smaller ionic radius of Tb(III).

*q_corr_* = *A’Δk_corr_* [*whereΔk_corr_* = (*k_H2O_* − *k_D2O_*)] (3)

whereas *k_H_*_2*O*_ and *k_D_*_2*O*_ are radiative rate constants in H_2_O and D_2_O solvent, *A’* is a proportionality constant signifying the sensitivity of the lanthanide ion to vibronic quenching by OH oscillators, and *q_corr_* is the hydration state, i.e., number of solvent molecules attached to a metal center.

**Figure 5:**
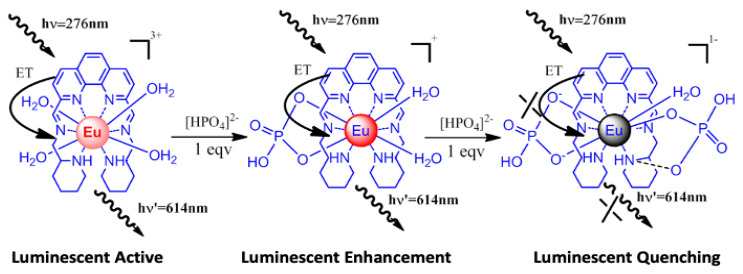
Proposed mechanistic pathway for successive HPO_4_^2−^ binding with **1** in aqueous medium.

To understand the underlying mechanism behind the titration profiles of **1** and **2** against the hydrogen phosphate ions (Figure 2a and Figure 3a), we performed time-resolved luminescence decay studies (Figures S18–S21) and also calculated the quantum yield of each complexes (Table S1). As a representative example lifetime was determined for **1** in the absence and presence of phosphate ions at different stoichiometry and compared with lifetime of the complex **1,** a laser excitation source of 276 nm was used, and the decay luminescence pattern was monitored at 614 nm. Aqueous (H_2_O as well as D_2_O) HEPES buffer solutions of **1** were used for these studies. In the absence of HPO_4_^2−^, luminescence profiles for **1** could be best fitted to single exponential decay traces with lifetime values τ = 0.22 ms (H_2_O) and 1.56 ms (D_2_O) (*κ*^2^ = 1.16, 1.18) (Figure S19, Table 1). Upon the addition of one equivalent HPO_4_^2−^ to these two solutions, the luminescence decay profiles could be best fitted to τ = 0.41 ms (H_2_O) and 1.72 ms (D_2_O) (*κ*^2^ = 1.17, 1.13) (Table 2). Thus, the decrease in the hydration state of **1** from *q* = 4 to *q* = 2 upon the addition of 1 equivalent of HPO_4_^2−^ (i.e., upon 1:1 association) was obvious to understand. After the addition of another equivalent of HPO_4_^2−^ to this mixture, the lifetime values were found to be 0.60 ms (H_2_O) and 1.89 ms (D_2_O) (*κ*^2^ = 1.20, 1.18) (Table 2), therefore revealing that hydration state *q* = 1, i.e., one coordinated water molecules present at 1:2 binding ratio between **1** and HPO_4_^2−^. The gradual addition of excess HPO_4_^2−^ did not change the hydration state of the resulting species in solution further, i.e., *q* = 1 (Table 2). Based on the results summarized in Table 2, a plausible mechanism of phosphate’s interaction with complex **1** is schematically represented in Figure 5. The initial little luminescence enhancement of **1** upon one equivalent HPO_4_^2−^ addition possibly arose due to the replacement of two coordinated water molecules by the incoming phosphate group, which normally functions as a strong chelating species [24]. It is noteworthy to mention that such an effect has already been observed in the case of bicarbonate [72]. The addition of a second equivalent of HPO_4_^2−^ to this 1:1 mixture resulted in the displacement of one more coordinated water molecule from the metal center (also supported by Figure S12) and shows quenching of luminescence. Possibly, steric crowding played an important role here by forcing the phosphate group to form a hydrogen bond with the piperidine NH moiety of ligand **L**, causing an energy mismatch between the lowest triplet state (T_1_) of **L** and the excited state of the Eu(III). Therefore, the energy transfer process from the ligand L to europium(III) terminated and hence resulted in quenching of the luminescence process. However, at higher concentrations, the emission intensity reduces completely, which can be ascribed to the leaching of lanthanides from the complexes in the presence of more strongly coordinating phosphates ions.

Although a significant number of luminescent complexes are applied in bio-imaging with excitation range below 300 nm [73,74], the presented complexes with excitation at 276 nm (i.e., in the UV region) limit their usage in in vivo bio-imaging. However, for in vitro conditions, the complexes are expected to be significant.

## 3. Conclusions

A series of mononuclear Ln(III) complexes (**1**–**3**) based on aminomethylpiperidine functionalized 1,10-phenanthrolene-based nitrogen-rich hexadentate heterocyclic ligand **L** has been reported. All these rare-earth complexes showed red-shifted metal-centered luminescence. The excited state photophysical properties of these complexes were explored to find their specific recognition affinity towards various important anions. The selective sensing of **1**–**3** for hydrogen phosphates over other anions is remarkable. Systematic spectrophotometric analysis demonstrates that, in the case of **1** and **2**, the emission intensities were increased slightly at the very beginning of phosphate addition (up to 1 and 2 equivalents, respectively) and finally decreased to a plateau at high phosphate concentrations (at mM level). The limits of detection (LOD) fall in the range 0.1–0.4 μM. Luminescence decay studies revealed that successive replacement of weakly bound coordinated water molecules from Eu(III) and Sm(III) probably caused the initial emission enhancement of these two complexes upon hydrogen phosphate addition. However, the addition of excess hydrogen phosphate causing steric crowding at the metal site and possible hydrogen bond formation between the piperidine NH group and phosphate might have created an energy mismatch between the lowest triplet state (T_1_) of **L** and the excited state of the Eu(III), which in turn resulted in termination of the energy transfer between the *o*-phenanthrolene moiety and Ln(III). 

## 4. Experimental Section

### 4.1. Materials and Methods

All chemicals were purchased from Aldrich. Sodium salts of all anions were used in this study. Elemental analyses of the complexes were carried out by using a vario Micro cube from Elementar. IR spectra were recorded from KBr pellets (1% *w*/*w*) on a Perkin–Elmer spectrum GX FTIR spectrophotometer. Electronic spectra were recorded on a Shimadzu UV 3600 spectrophotometer and scanned in the range 200–800 nm. The mass-spectrometric analysis was performed by using the positive ESI technique on a Waters Q-ToF Micromass spectrometer in CH_3_OH. NMR spectra were recorded on a Bruker *Avance* 500 MHz FT-NMR spectrometer. The chemical shifts (δ) for proton resonances are reported in ppm relative to the internal standard TMS (Tetramethylsilane). The CD spectra were recorded by using a JASCO 815 spectrometer. Milli-Q water was used as a solvent. pH measurements were carried out using an ORION VERSA STAR pH meter. Emission spectra were recorded using an Edinburgh Instruments model Xe-900, and all the spectra recorded are reported hereafter applying emission correction. The slit sizes for emission and excitation were adjusted as 3.0/3.0 nm. For Job plot analysis (continuous variations method), a series of samples were prepared with a constant sum of concentrations at 1.0 μM but with varying concentrations of complex and hydrogen phosphate. The luminescence spectra were recorded for each sample with λ_ex_ = 276 nm for all these complexes. The maximum luminescence intensity was plotted versus the mole fraction of the corresponding hydrogen phosphate. For determination of the maximum, the ascending and descending segments of the curve were fitted to linear lines, respectively, and the intercept of both lines denotes the maximum and thus the stoichiometry of the complex.

**Synthesis of ligand L. 4(H_2_O)**: 1,9-Diformyl-1,10-phenanthroline (0.001 mmol, 0.200 g) (*17*) was dissolved in 50 mL of CH_3_OH. To this methanolic solution, 2-(aminomethyl)piperidine (0.002 mmol, 0.184 g) was added drop by drop. This reaction mixture was stirred continuously for 48 h at 50 °C. During this, the color of the reaction mixture changed to red-brown, indicating the formation of a Schiff base. The solvent was removed under vacuum, and the resultant orange-red powder was isolated. Yield. 70%. -^1^H NMR (CDCl_3_, 500 MHz): δ = 8.26, 8.24 (*dd*, *J* = 3 Hz, 2H), 7.86, 784, 7.82(*t*, *J* = 10 Hz, 4H), 7.77(*s*, 2H), 3.55(*brs*, 2H), 3.27(*m*, 2H), 3.09, 3.07, 3.05, 3.03 (*q*, *J* = 10 Hz,2H), 2.83, 2.81, 2.79 (*t*, *J* = 12 Hz, 2H), 2.50 (*m*, 2H), 2.24, 2.22, 2.20 (*t*, *J* = 11 Hz, 2H), 1.93–1.83(*dd*, *J* = 12 Hz, 4H), 1.60–1.50(*m*, 6H), 1.34–1.31 (*m*, 2H). ^13^C NMR, (CDCl_3_, 125. MHz) δ = 160.22, 145.27, 136.89, 128.66, 126.30, 122.75, 122.64, 83.14, 83.06, 64.20, 50.66, 48.99, 28.64, 24.91, 23.95. DEPT-135°. 131.59, 121.00, 117.44, 117.34, 77.85, 58.93 (CH, UP), 45.40, 43.72, 23.37, 19.64, 18.68 (CH_2_, DOWN). IR (KBr): *υ* cm^−1^ = 3418 (br), 1616 (s), 1598 (s), 1370 (s). UV vis (CH_3_OH, nm (ε, M^−1^ cm^−1^)): λ = 275 (32170), 234(31530); ESI[MS]^+^ in methanol: *m*/*z* (calcd (found)) 429.28 (429.69 for **L**+H^+^; 100% abundance); 451.26 (451.69 for **L**+Na^+^; 90% abundance). Elemental data: Calc (found) for C_26_H_32_N_6_.4H_2_O: C 62.38 (62.44), H 8.05 (7.74), N 16.79 (16.22)%.

### 4.2. Synthesis of Complexes

**General synthetic procedure for compounds 1**–**3**. The methanolic solution of the ligand **L** (0.001 mmol) and LnCl_3_ salt (0.001 mmol) was mixed together and allowed for constant stirring at room temperature for 4 h. After completion of the reaction, the solution was evaporated by rotary and the solid was further dried under vacuum.

**Complex 1**. Yield: 75%. IR (KBr): *υ* cm^−1^ = 3398 (br), 1623 (s), 1458 (m), 1432 (m) 1400 (s), UV vis (HEPES Buffer, pH 7.4, nm (ε, M^−1^ cm^−1^)): λ = 237 (21,392), 285 (20,212); ESI-[MS]^+^ in methanol: *m*/*z* calcd(found) 687.10 (687.12) for ([Eu**L**(Cl)_3_+H^+^]; 65% abundance), 651.13(651.15) for ([Eu**L**(Cl)_2_]^+^; 100% abundance). Elemental data: Calc (found) for C_26_H_70_Cl_3_EuN_6_O_19,_ C 30.34(30.54), H 6.86 (6.51), N 8.17 (8.24)%. 

**Complex 2**. Yield: 68%. IR (KBr): *υ* cm^−1^ = 3436 (br), 1629 (s), 1459 (m), 1431 (m) 1386 (s), -UV vis (HEPES Buffer, pH 7.4, nm (ε, M^−1^ cm^−1^)): λ = 236 (16,825), 287 (14,175); ESI-[MS]^+^ in methanol: *m*/*z* calcd(found) 706.27 (706.21) for ([Sm(**L-2H).4**(CH_3_OH)]^+^; 100% abundance); 770.33 (770.27) for ([Sm**L-2H).6**(CH_3_OH)]^+^; 100% abundance). Elemental data: Calc (found) for C_26_H_66_Cl_3_N_6_O_17_Sm: C 31.49(31.11), H 6.71(6.65), N 8.48(8.54)%.

**Complex 3**. Yield 65%. IR (KBr): *υ* cm^−1^ = 3432 (br), 1627(s), 1459 (m), 1432 (m) 1390 (s). -UV vis (HEPES Buffer, pH 7.4, nm (ε, M^−1^ cm^−1^)): λ = 236 (215,725), 285 (13,917); ESI-[MS]^+^ in methanol: *m*/*z* calcd(found) 693.11.(693.09) for ([Tb**L**(Cl)_3_+H^+^]; 90% abundance); 657.13 (657.12) for ([Tb**L**(Cl)_2_]^+^; 100% abundance). Elemental data: Calc (found) for C_26_H_70_Cl_3_N_6_O_19_Tb: C 30.14(29.68), H 6.81(7.05), N 8.11(8.34)%.

Detection limit (DL) calculation:

DL = CL × ET(4)

where DL = detection limit, CL = Concentration of complex, and ET = Equivalent of Titrant at which change was observed. Here, the titrant is phosphate.

## Figures and Tables

**Table 1:** Excited state lifetime measurements of **1**–**3** in H_2_O and D_2_O at pH 7.4 (HEPES buffer).

Complex	τH_2_O (ms)	τD_2_O(ms)	*q_corr_* *^a^*	Coordination Number
**1**	0.22	1.56	4.38	10
**2**	0.15	1.11	3.70 *	10
**3**	0.47	0.73	3.50	9

*^a^ q_corr_* values were determined by adapting *A’* = 1.2 ms (Eu^3+^) and 5 ms (Tb^3+^) and *Δk_corr_* = −0.25 ms^−1^ (Eu^3+^) and −0.06 ms^−1^ (Tb^3+^). * For Sm(III), since *Δk_corr_* values are not available in the literature, the *q_corr_* value for complex **2** is calculated without applying the correction.

**Table 2:** Summary of the changes in the lifetimes of complex **1** in the presence of different equivalent HPO_4_^2−^ in aqueous media (H_2_O as well as D_2_O).

Species	1 + HPO_4_^2^^−^ (1:1)	1 + HPO_4_^2^^−^ (1:2)	1 + HPO_4_^2^^−^ (1:10)
**τ** **(H_2_O) (ms)**	0.41	0.60	0.62
**τ** **(D_2_O) (ms)**	1.72	1.90	1.91
***q*** **_*corr*_**	1.98	1.12	1.06

## Data Availability

Data used to support the findings of this study are included within the article and supporting material.

## Supplementary Materials

The following are available online at https://www.mdpi.com/2079-4991/11/1/53/s1, Figure S1: ^1^H NMR of **L** in CDCl_3_, Figure S2: ^13^C NMR of **L** in CDCl_3_, Figure S3: DEPT-135° NMR of **L** in CDCl_3_, Figure S4: ESI-MS Spectrum of **L**, Figure S5:CD spectra of Ligand **L** in CHCl_3_, Figure S6: IR-Spectra of **L**, **1**, **2**, and **3**, Figure S7: ESI-MS Spectrum of **1**, Figure S8: ESI-MS Spectrum of **2**, Figure S9: ESI-MS Spectrum of **3**, Figure S10: Normalization Spectra, Figure S11: Emission Curve of **1** against HPO_4_^2^^−^, Figure S12: ESI-MS spectrum of [1]:2[HPO_4_^2^^−^], Figure S13: Non-linear fit curve of **1** against HCO_3_^−^, Figure S14: Emission curve of **2** against HPO_4_^2^^−^, Figure S15: ESI-MS spectrum of [2]:2[HPO_4_^2^^−^], Figure S16: Emission curve of **3** against HPO_4_^2^^−^, Figure S17: ESI-MS spectra of [3]:2[HPO_4_^2^^−^], Figure S18: (a) UV-vis spectra of ligand **L** and its complexes **1**, **2** and **3** and (b) possible energy transfer, Figure S19: Excited state lifetime of complex **1** with HPO_4_^2^^−^ with 1:1 ratio, Figure S20: Excited state lifetime of complex **1** with HPO_4_^2^^−^ with 1:2 ratio, Figure S21: Excited state lifetime of complex **1** with HPO_4_^2^^−^ with 1:10 ratio, Table S1: Quantum yield calculation for complex **1**, **2** and **3**.
