# Supplementary material for: Mechanistic Insight of Sensing Hydrogen Phosphate in Aqueous Medium by Using Lanthanide(III)-Based Luminescent Probes
Source: Nanomaterials (Basel). 2020 Dec 28;11(1):53. doi: 10.3390/nano11010053 (PMC7824681; doi:10.3390/nano11010053)
Supplement: Supplementary file 1 [file nanomaterials-11-00053-s001.pdf]

Electronic Supplementary Material

Mechanistic Insight of Sensing Hydrogen Phosphate in Aqueous Medium by Using Lanthanide(III)-Based Luminescent Probes

Jashobanta Sahoo [1,2,3], Santlal Jaiswar [4], Pabitra B. Chatterjee [2,5], Palani S. Subramanian [1,2,*] and Himanshu Sekhar Jena [6,*]

[1] Inorganic Materials and Catalysis Division, Central Salt and Marine Chemicals Research Institute, (CSIR-CSMCRI), Bhavnagar, Gujarat 364 002, India;
[2] Academy of Scientific and Innovative Research (AcSIR), CSIR-CSMCRI, Bhavnagar, Gujarat 364 002, India; pbchatterjee@csmcri.res.in
[3] Department of Chemistry, Hindol College, Khajuriakata, Higher Education Department, State Government of Odisha, Bhubaneswar- 751001, Odisha, India
[4] Discipline of Marine Biotechnology and Ecology, CSIR-CSMCRI, Bhavnagar, Gujarat 364 002, India; santlal@csmcri.res.in
[5] Analytical Discipline and Centralized Instrument Facility, CSIR-CSMCRI, Bhavnagar, Gujarat 364 002, India
[6] Department of Chemistry, Institution: Ghent University, Krijgslaan 281-S3 B, 9000 Ghent, Belgium
* Correspondence: siva140@yahoo.co.in or siva@csmcri.org (P.S.S.); hsjena@gmail.com or Himanshu.jena@ugent.be (H.S.J.)

Citation: Sahoo, J.; Jaiswar, S.; Chatterjee, P.B.; Subramanian, P.S.; Jena, H.S. Mechanistic Insight of Sensing Hydrogen Phosphate in Aqueous Medium by Using Lanthanide(III) Based Luminescent Probes. *Nanomaterials* **2021**, *11*, 53. https://doi.org/10.3390/nano11010053

Publisher's Note: MDPI stays neutral with regard to jurisdictional claims in published maps and institutional affiliations.

Copyright: © 2020 by the authors. Submitted for possible open access publication under the terms and conditions of the Creative Commons Attribution (CC BY) license (http://creativecommons.org/licenses/by/4.0/).

Contents: **Page Number**

Figure S1. ^{1}H NMR of **L** in $CDCl_3$--S3

Figure S2. ^{13}C NMR of **L** in $CDCl_3$---S3

Figure S3. DEPT-135° NMR of **L** in $CDCl_3$--S4

Figure S4. ESI-MS Spectrum of **L**---S4

Figure S5. CD spectra of Ligand **L** in $CHCl_3$--S5

Figure S6. IR-Spectra of **L, 1, 2**, **and 3**---S5

Figure S7. ESI-MS Spectrum of **1**--S6

Figure S8. ESI-MS Spectrum of **2**--S6

Figure S9. ESI-MS Spectrum of **3**--S7

Figure S10. Normalization Spectra---S7

Figure S11. Emission Curve of **1** against HPO_4^{2-} --S8

Figure S12. ESI-MS spectrum of [**1**]:2[HPO_4^{2-}] ---S8

Figure S13. Non-linear fit curve of **1** against HCO_3^{-} --S9

Figure S14. Emission curve of **2** against HPO_4^{2-} --S9

Figure S15. ESI-MS spectrum of [**2**]:2[HPO_4^{2-}] --S10

Figure S16. Emission curve of **3** against HPO_4^{2-} --S10

Figure S17. ESI-MS spectra of [**3**]:2[HPO_4^{2-}] --S11

Figure S18 (a) UV-vis spectra of ligand L and its complexes **1**, **2** and **3**

and (b) possible energy transfer--S11

Figure S19. Excited state lifetime of complex **1** with HPO_4^{2-} with 1:1 ratio---------------------------- S12

Figure S20. Excited state lifetime of complex **1** with HPO_4^{2-} with 1:2 ratio-----------------------------S12

Figure S21. Excited state lifetime of complex **1** with HPO_4^{2-} with 1:10 ratio---------------------------- S13

Table S1: Quantum yield calculation for complex **1** ,**2** and **3**--S13

Figure S1. ^{1}H NMR of **L** in $CDCl_3$

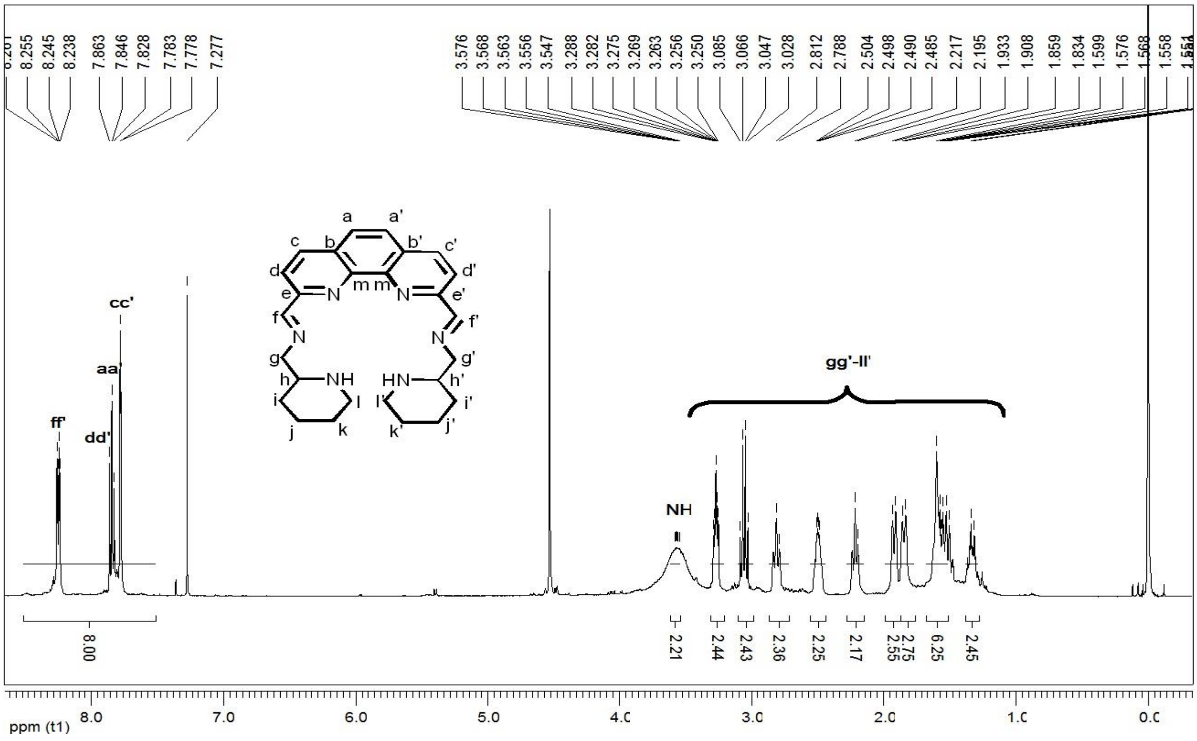

Figure S2. ^{13}C NMR of **L** in $CDCl_3$

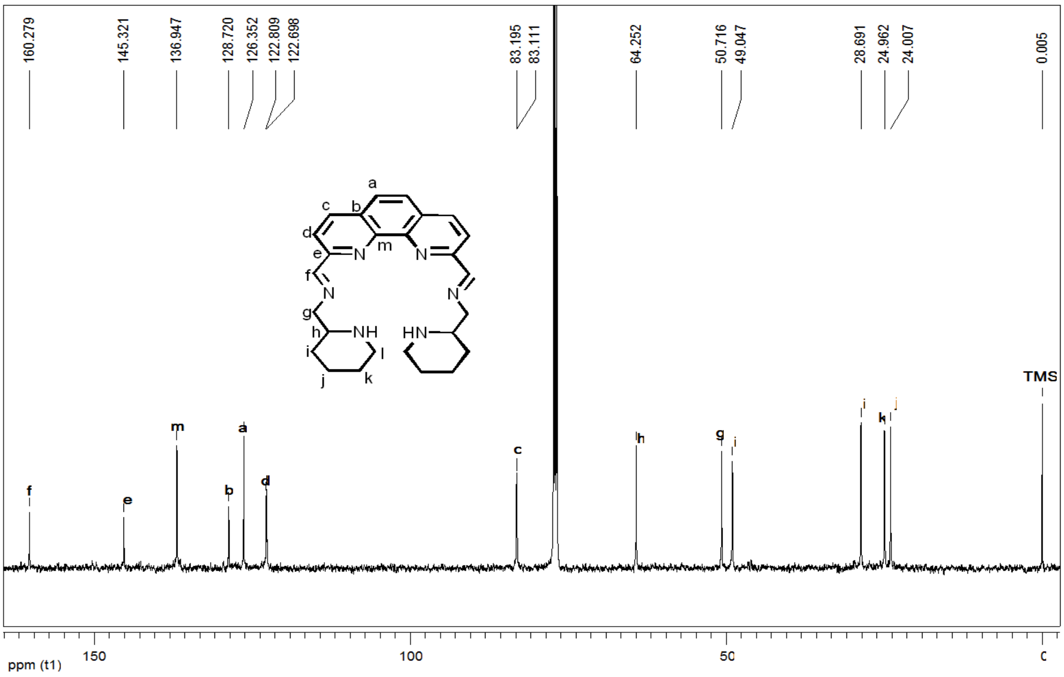

Figure S3. DEPT -135° NMR of **L** in $CDCl_3$

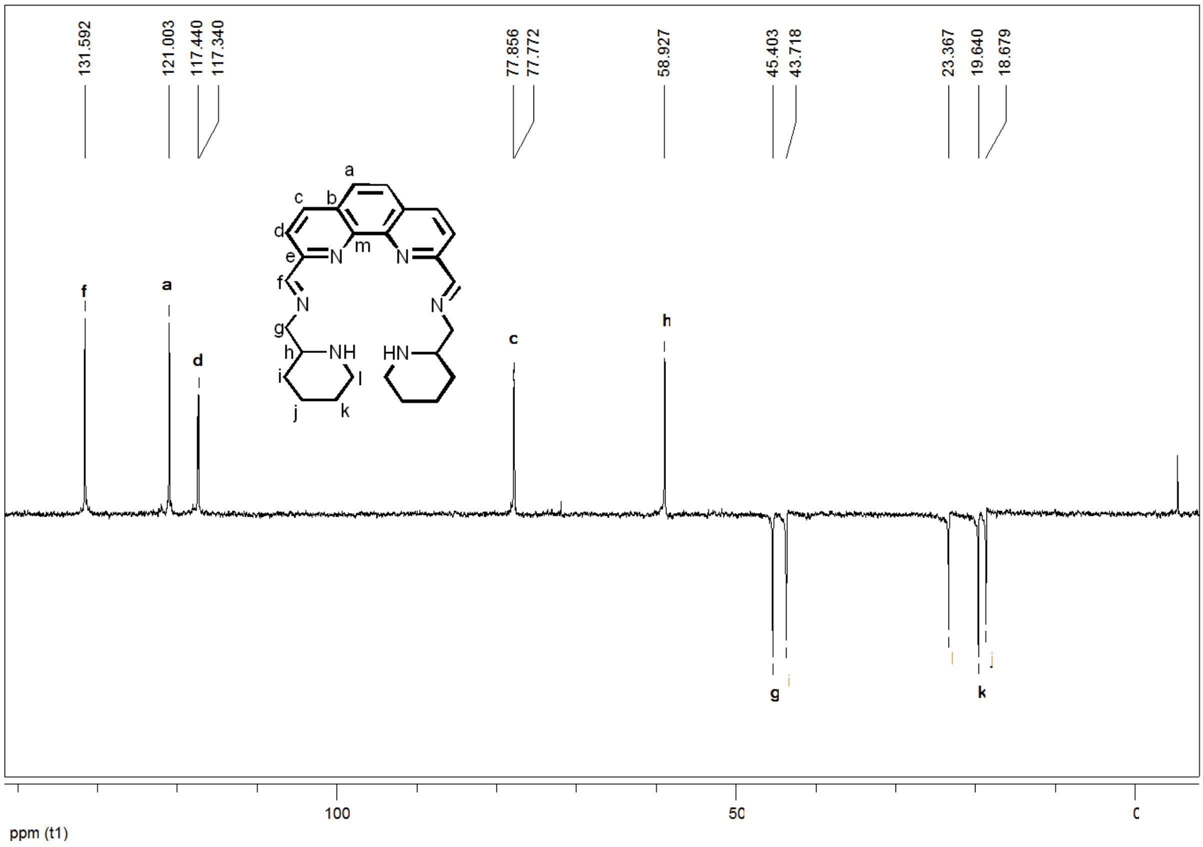

Figure S4. ESI-MS spectrum of **L** recorded in methanol.

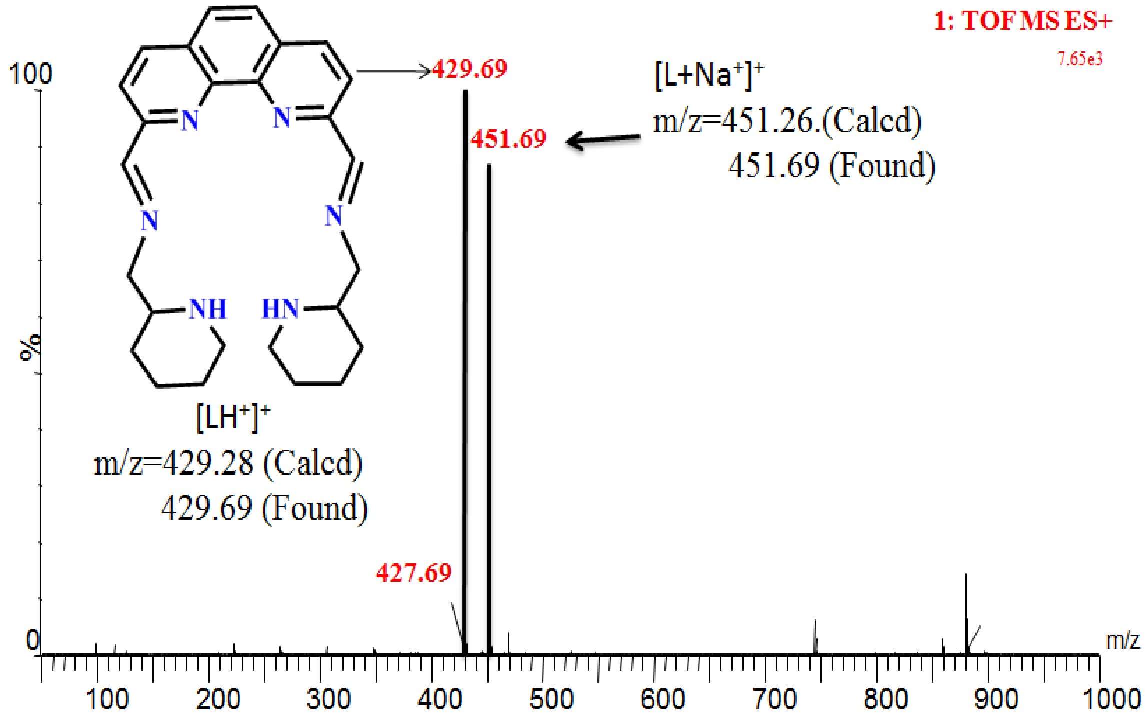

Figure S5. CD spectra of Ligand **L** in $CHCl_3$

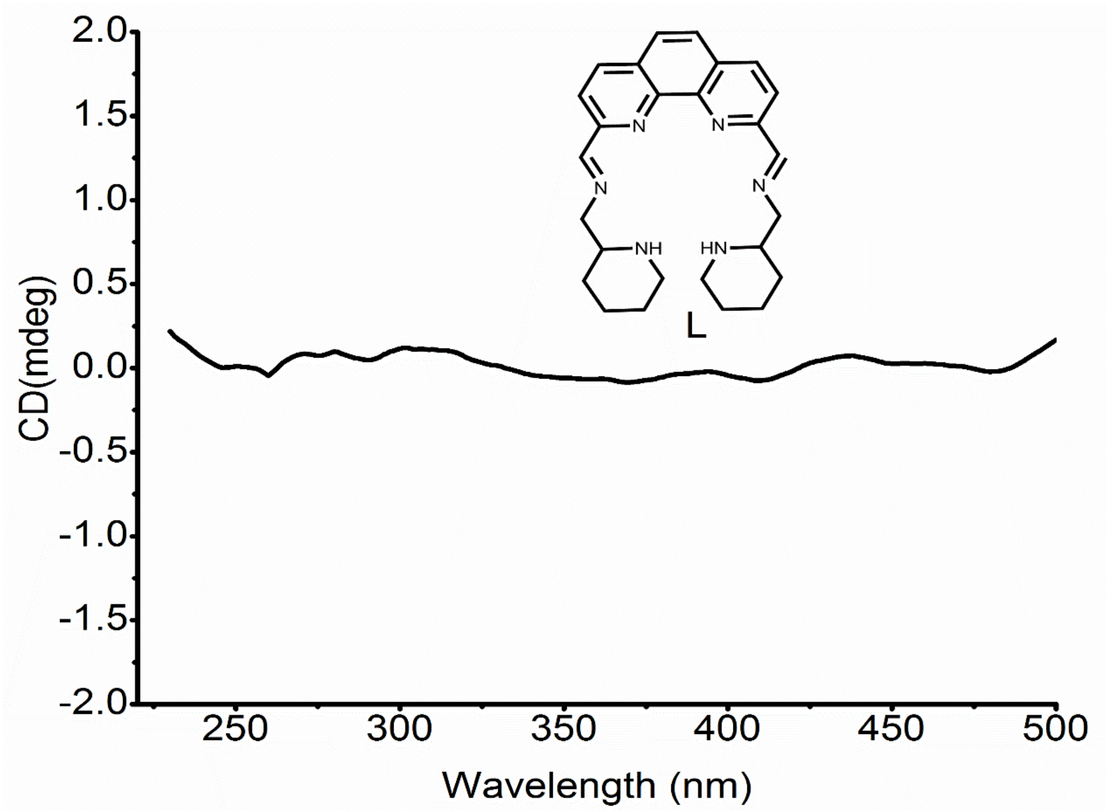

Figure S6. IR-Spectra of **L, 1, 2** and **3**

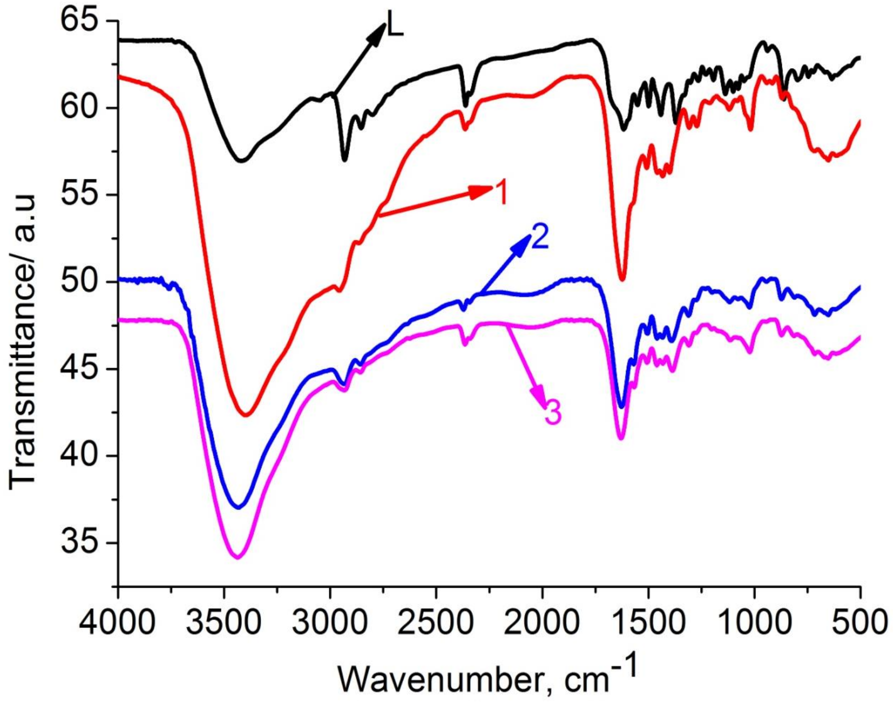

Figure S7. ESI-MS spectrum of **1** recorded in methanol.

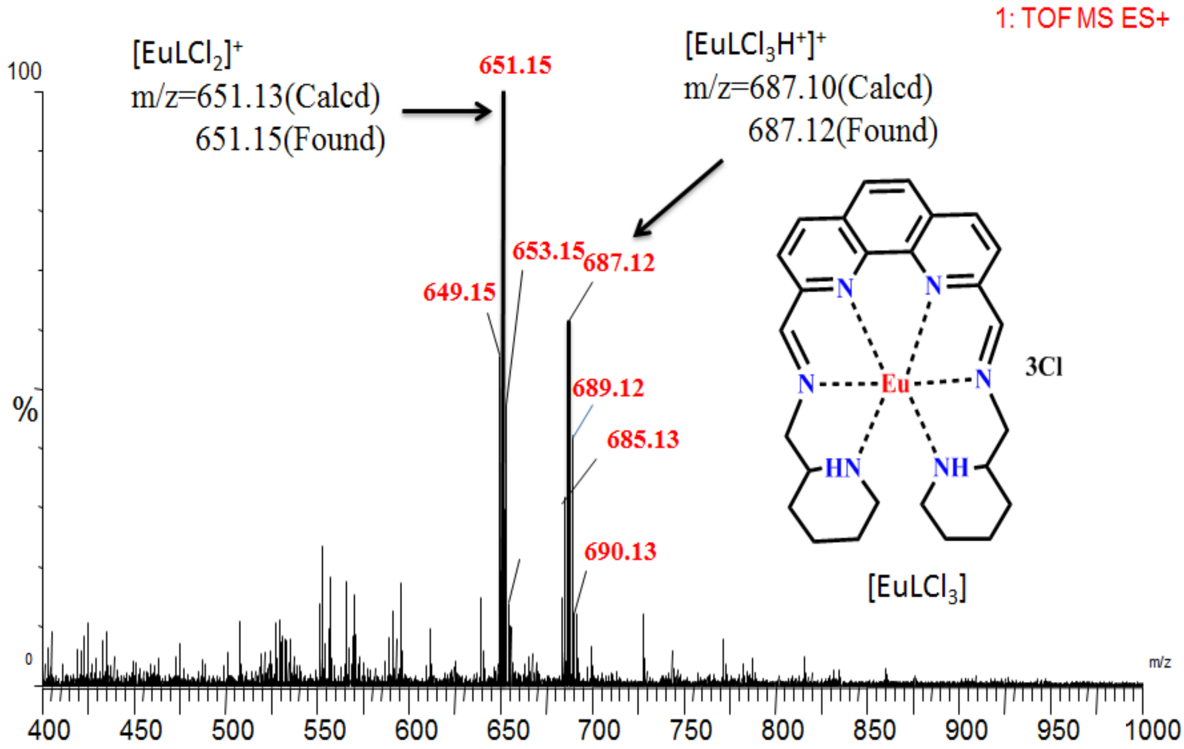

Figure S8. ESI-MS spectrum of **2** recorded in methanol.

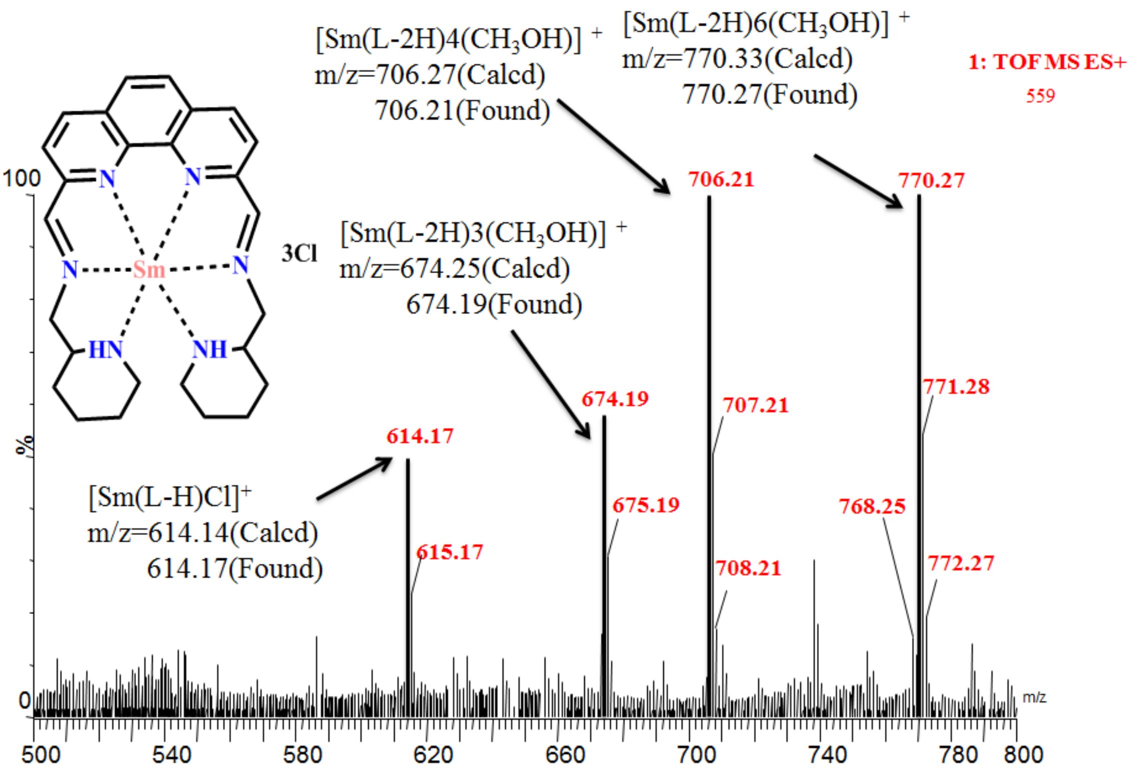

Figure S9. ESI-MS spectrum of **3** recorded in methanol.

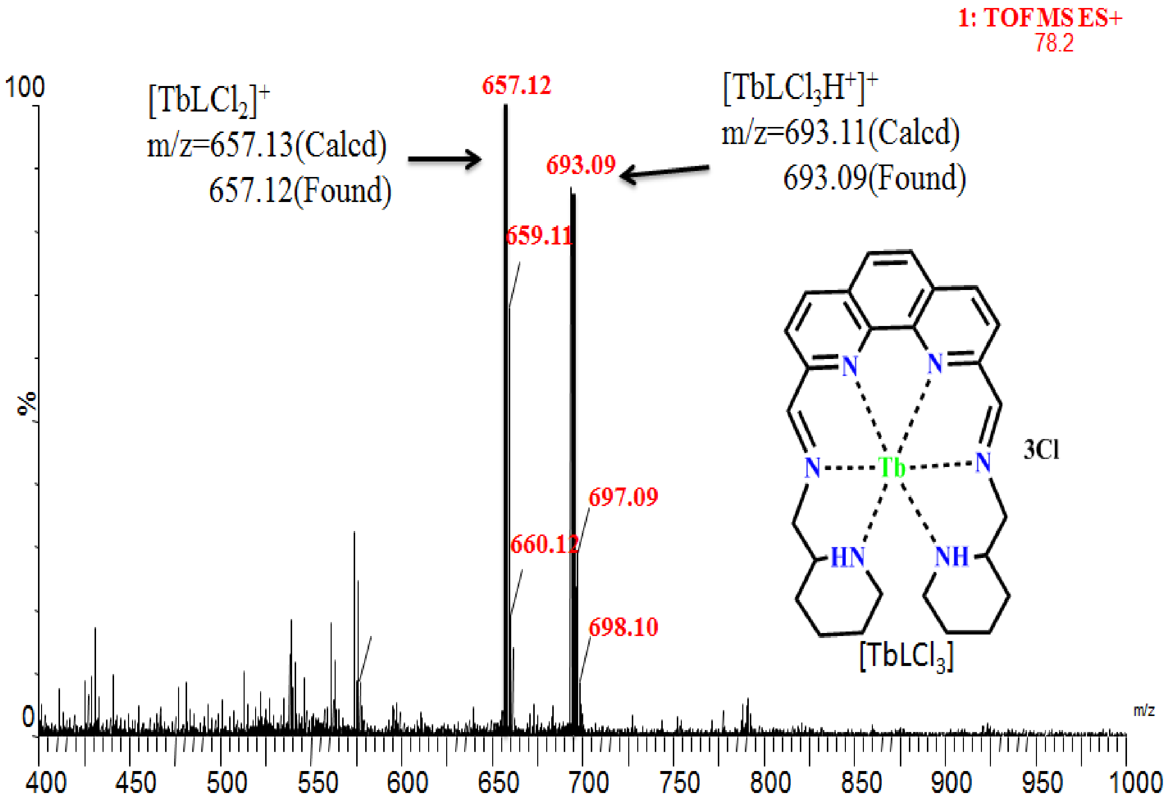

Figure S10. Normalization Spectra of **1**, **2** and **3**

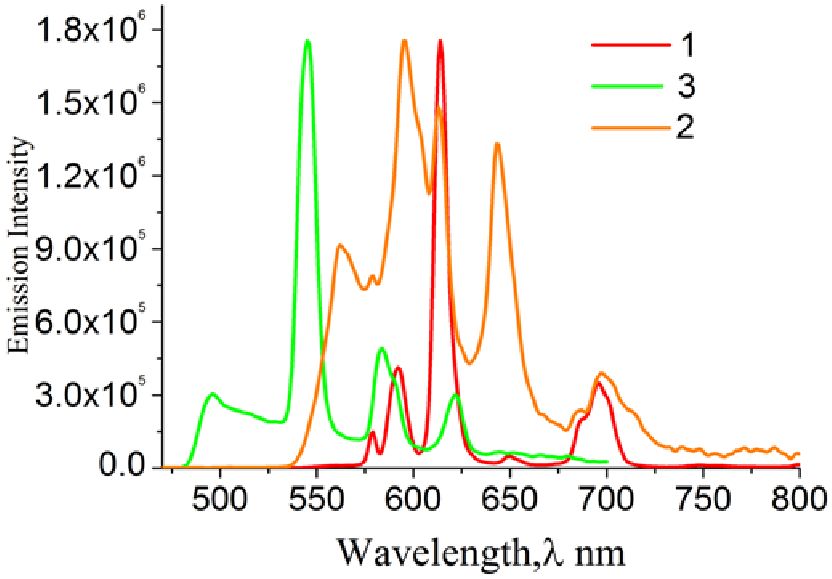

Figure S11. Emission spectra of **1** against varying the concentration of HPO_4^{2-} ranging from 0.01-400 equivalents.

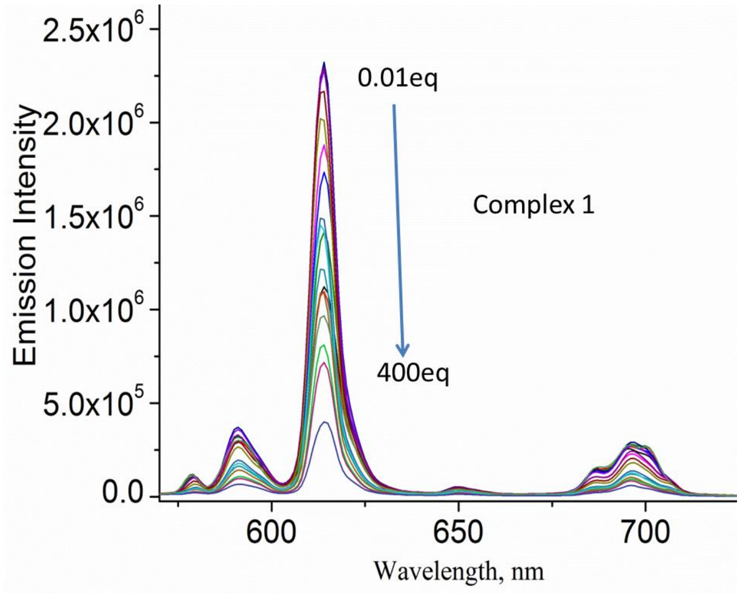

Figure S12. ESI-MS spectrum of **[1]:2[**HPO_4^{2-}**]** in water.

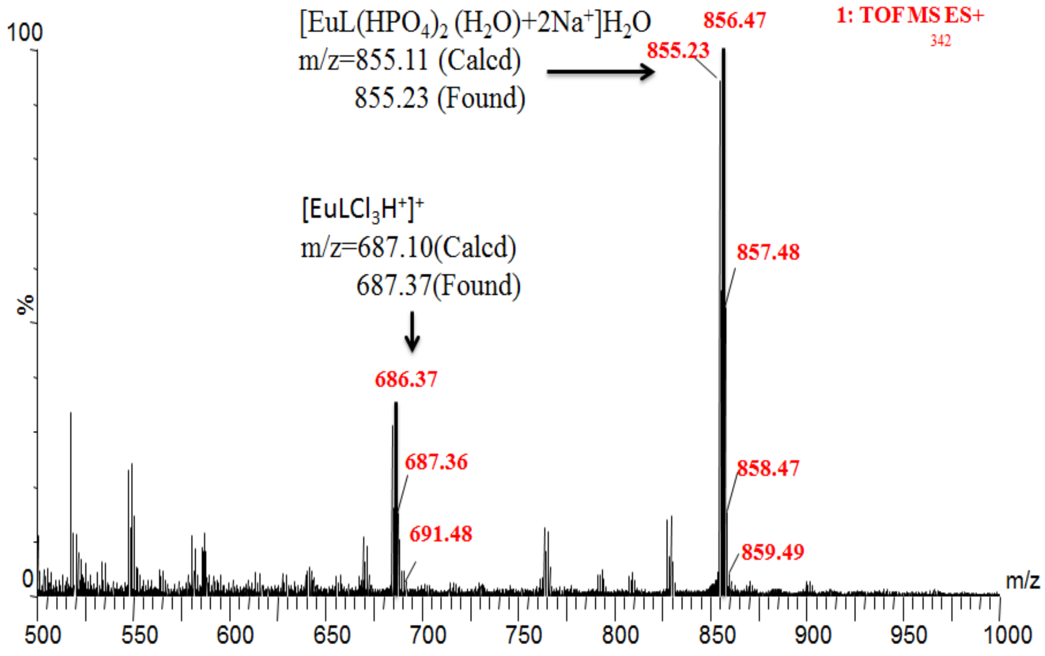

Figure S13. Nonlinear curve of **1** against HCO_3^- (10-600 equiv)

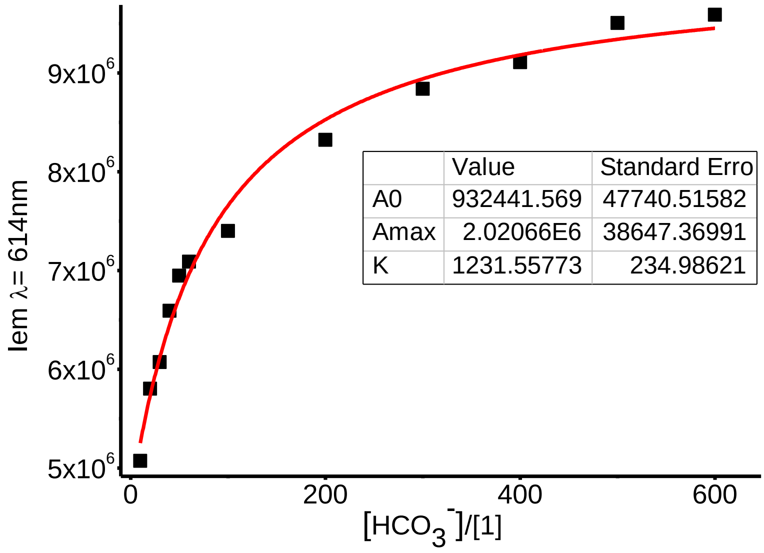

	Value	Standard Erro
A0	932441.569	47740.51582
Amax	2.02066E6	38647.36991
K	1231.55773	234.98621

Figure S14. Emission spectra of 1 against varying the concentration of HPO42- ranging from 0.01-800 equivalents.

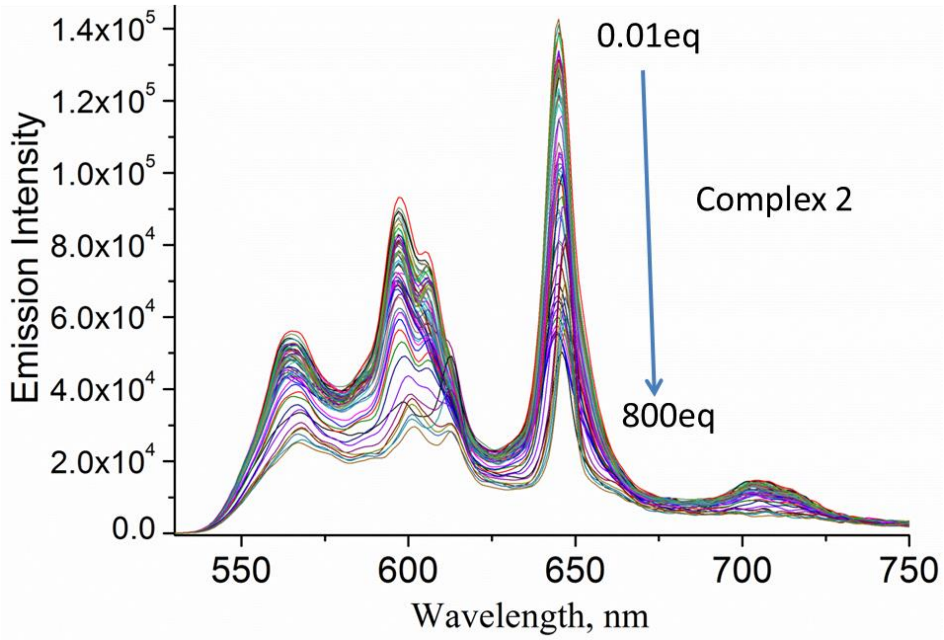

Figure S15. ESI-MS spectrum of **[2]:2[**HPO_4^{2-}**]** in water.

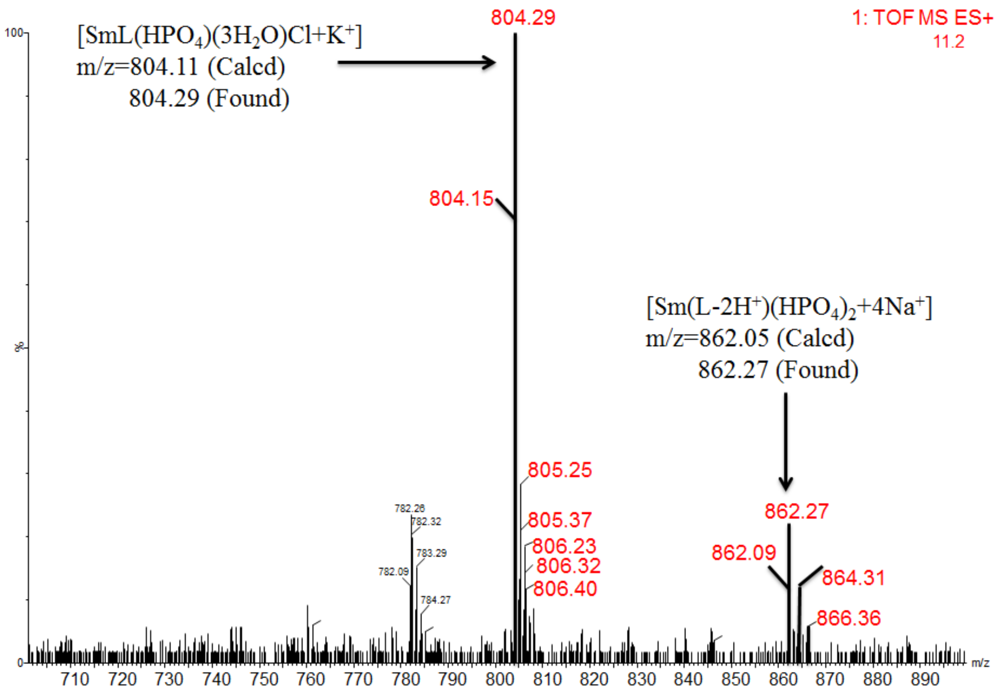

Figure S16. Emission spectra of 1 against varying the concentration of HPO42- ranging from 0.01-5equivalents.

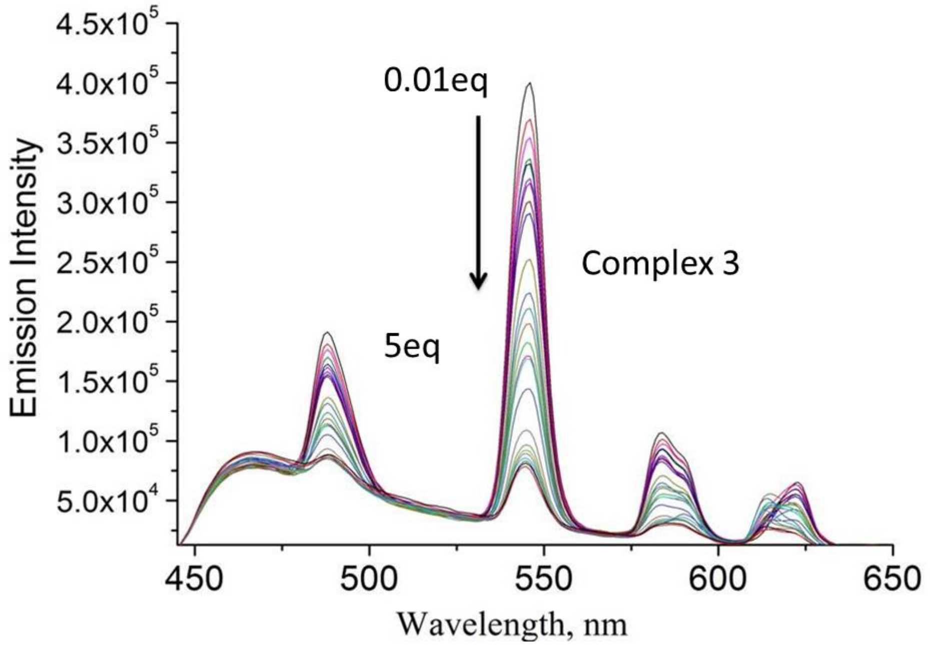

Figure S17. ESI-MS spectrum of **[3]:2[**HPO_4^{2-}**]** in water.

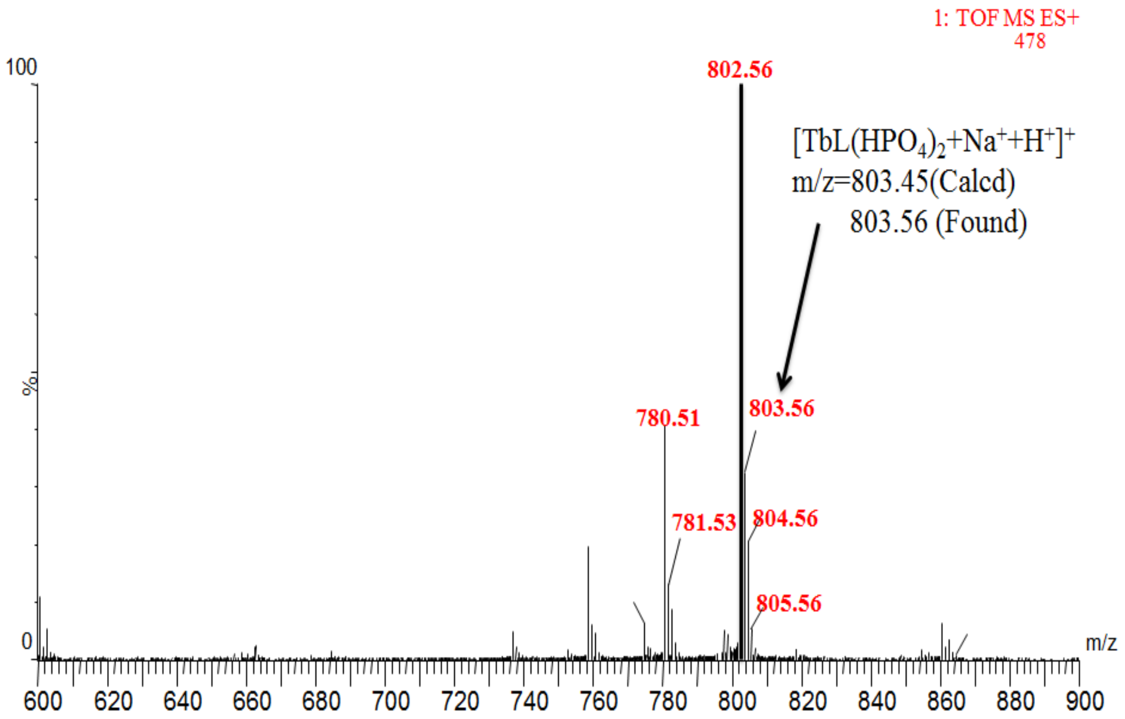

Figure S18. (a) UV-vis spectra of ligand L and its complexes 1, 2 and 3 (2x10-5M) in HEPES buffer pH 7.4, (b) scheme to represent the possible energy transfer during this process.

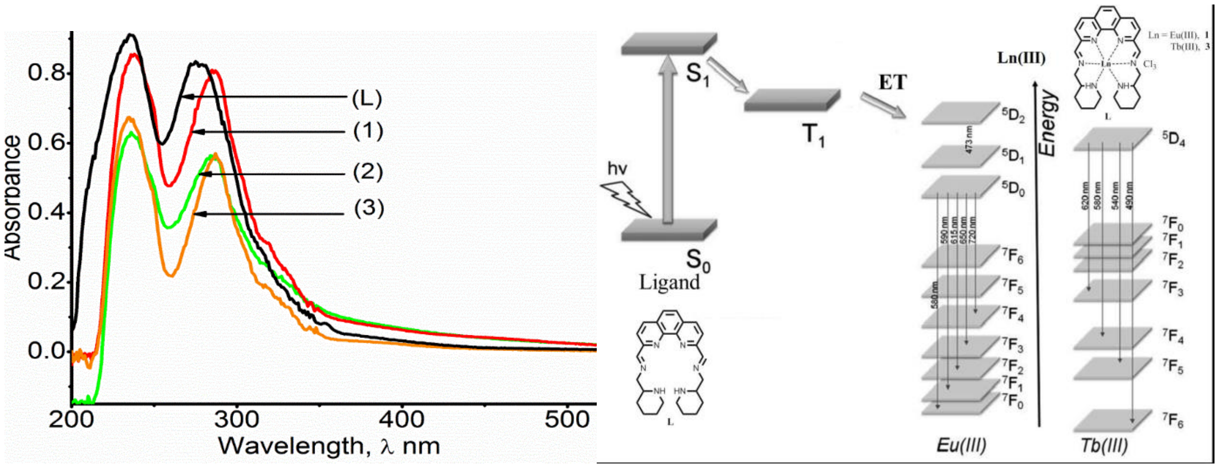

(a) (b)

Figure S19. Exited state lifetime of complex 1 with HPO_4^{2-} with 1:1 ratio.

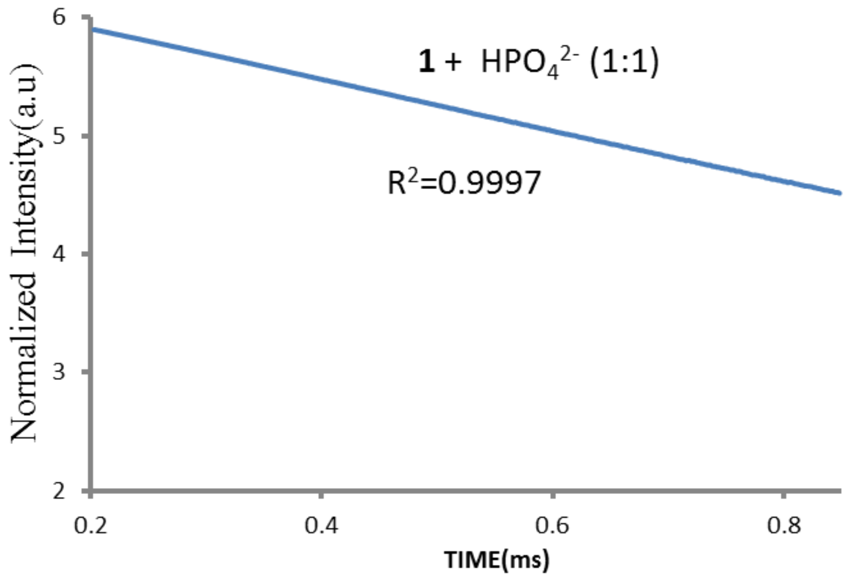

Figure S20. Exited state lifetime of complex 1 with $HPO_4^{2-}$with 1:2 ratio.

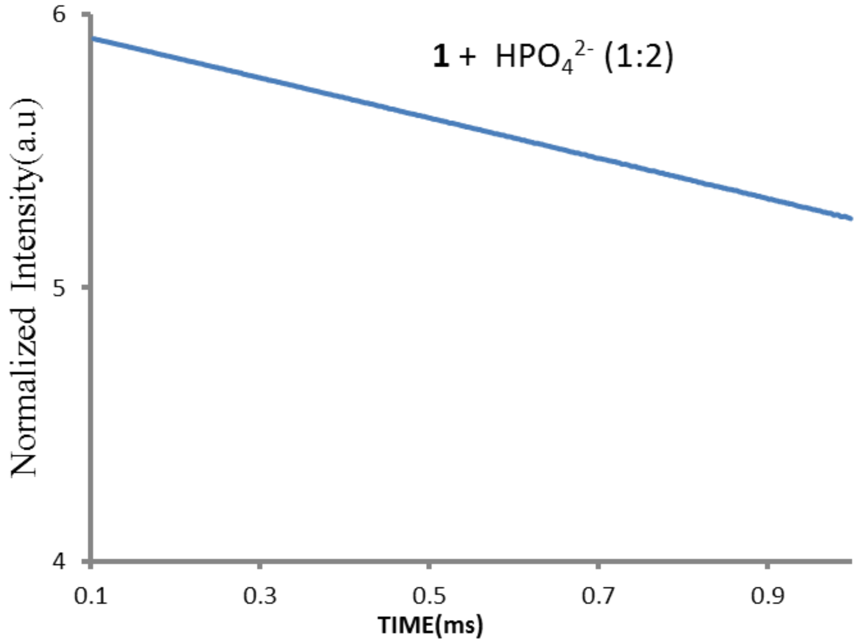

Figure S21. Exited state lifetime of complex 1 with HPO_4^{2-} with 1:10 ratio.

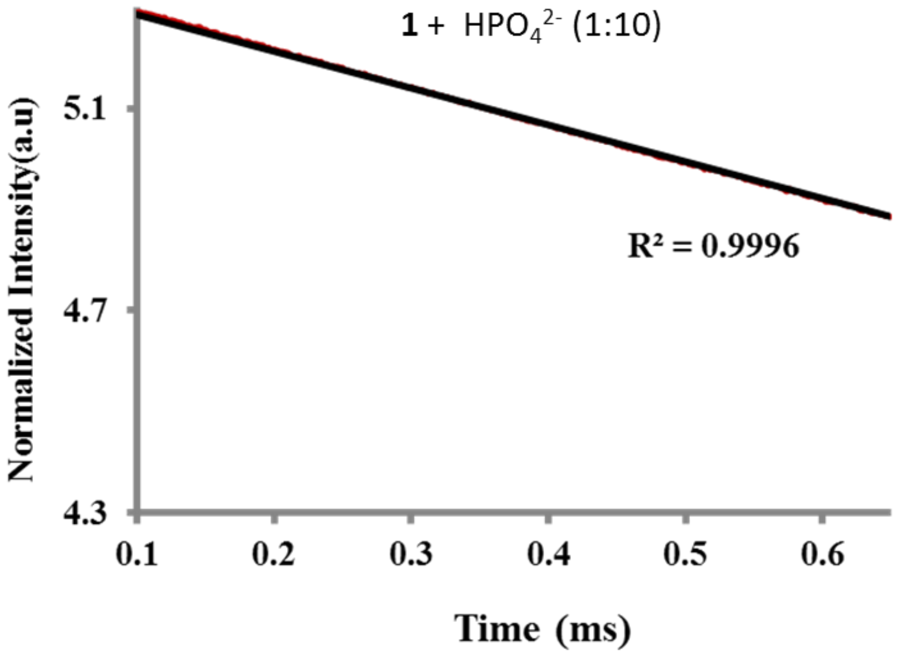

Table S1: Quantum yield calculation for complex 1 ,2 and 3.

Complex	Quantum yield[1]*(Φ)* (%)
1	12
2	7
3	5

[1]Bünzli J-CG, Chauvin A-S, Kim HK, Deiters E, Eliseeva SV (2010) Lanthanide luminescence efficiency in eight- and nine-coordinate complexes: Role of the radiative lifetime. Coord Chem Rev 254:2623-2633.
